# Anisodamine Ameliorates Hyperkalemia during Crush Syndrome through Estradiol-Induced Enhancement of Insulin Sensitivity

**DOI:** 10.3389/fphar.2019.01444

**Published:** 2019-11-29

**Authors:** Jian-Guang Yu, Bo-Shi Fan, Jin-Min Guo, Yun-Jie Shen, Ye-Yan Hu, Xia Liu

**Affiliations:** ^1^Department of Pharmacy, Shanghai Chest Hospital, Shanghai Jiao Tong University, Shanghai, China; ^2^Department of Pharmacology, Second Military Medical University, Shanghai, China; ^3^Department of Thoracic Surgery, Sixth Medical Center of PLA General Hospital, Beijing, China; ^4^Department of Pharmacy, 960 Hospital of the Joint Logistics Support Force of the Chinese People’s Liberation Army, Jinan, China

**Keywords:** anisodamine, estradiol, α7 nicotinic acetylcholine receptor, crush syndrome, hyperkalemia, insulin sensitivity

## Abstract

Hyperkalemia is a major cause of on-site death in crush syndrome (CS), which is more severe and common in male victims. Anisodamine is a belladonna alkaloid and widely used in China for treatment of shock through activation of α7 nicotinic acetylcholine receptor (α7nAChR). The present work was designed to study the protective effect of anisodamine in CS and the possible role of estradiol involved. Male and ovariectomized female CS mice exhibited lower serum estradiol and insulin sensitivity, and higher potassium compared to the relative female controls at 6 h after decompression. There was no gender difference in on-site mortality in CS mice within 24 h after decompression. Serum estradiol increased with similar values in CS mice of both gender compared to that in normal mice. Anisodamine decreased serum potassium and increased serum estradiol and insulin sensitivity in CS mice, and methyllycaconitine, selective antagonist of α7nAChR, counteracted such effects of anisodamine. Treatment with anisodamine or estradiol increased serum estradiol and insulin sensitivity, decreased serum potassium and on-site mortality, and eliminated the difference in these parameters between CS mice received ovariectomy or its sham operation. Anisodamine could also increase blood pressure in CS rats within 3.5 h after decompression, which could also be attenuated by methyllycaconitine, without influences on heart rate. These results suggest that activation of α7nAChR with anisodamine could decrease serum potassium and on-site mortality in CS through estradiol-induced enhancement of insulin sensitivity.

## Introduction

Crush syndrome (CS), due to compression of the extremities or other parts of the body, is characterized by rhabdomyolysis-induced metabolic disorders (e.g. hyperkalemia), hypovolemic shock, and acute kidney injury, etc. (Better, 1997; Oda et al., 1997; Bywaters and Beall, 1998; Slater and Mullins, 1998; Sever et al., 2015). CS in great earthquakes, is the most common cause of death, apart from trauma (Ukai, 1997). Up to 20% victims suffering from CS died of cardiac arrest caused by hyperkalemia or hypovolemic shock shortly after decompression (Ashkenazi et al., 2005), and hyperkalemia is the most important and fatal medical complication in CS patients. Male CS victims were characterized by higher serum potassium at admission and more frequently faced with fatal hyperkalemia after mass disasters (Sever et al., 2002; Sever et al., 2003; Sever et al., 2004). However, causes of gender difference in serum potassium during CS remain unclear, and drugs to safely reduce on-site mortality in CS, except for intravenous fluid resuscitation, are still clinically vacant (Sever and Vanholder, 2013).

Anisodamine (Ani) is a belladonna alkaloid isolated from the Chinese medicinal herb *Scopolia tangutica* Maxim of the Solanaceae family, and it has been used clinically for decades primarily to ameliorate circulatory disorders such as disseminated intravascular coagulation and septic shock. Our previous studies found that Ani could indirectly activate α7 nicotinic acetylcholine receptor (α7nAChR) to decrease serum potassium through enhancement of insulin sensitivity, resulting in decline of on-site mortality in CS, and Ani might be a promising on-site remedy for CS (Fan et al., 2016). Activation of a7nAChR was reported to increase serum estradiol (E_2_) level in ovariectomized rats (Ma et al., 2015). In addition, plenty of researches have demonstrated that E_2_ administration could ameliorate insulin resistance and enhance insulin sensitivity (Park et al., 2017; Qiu et al., 2018; Torres et al., 2018; Yan et al., 2019). Therefore, we speculated that gender difference in serum potassium during CS depends on level of serum E_2_; E_2_ could decrease serum potassium through elevation of insulin sensitivity, and even to decrease on-site mortality in CS.

The present work was designed to study the protective effect of Ani on serum potassium and on-site mortality in CS and the possible role of E_2_ involved. The influence of Ani on blood pressure after decompression in CS was also examined. Here, we show for the first time that activation of α7nAChR with Ani could increase serum E_2_ which further enhances insulin sensitivity to decrease serum potassium, contributing to the decline of on-site mortality in CS.

## Materials and Methods

### Animals and Reagents

C57BL/6 mice (22∼25 g, male; 20∼22 g, female) and Sprague-Dawley rats (230∼270 g, male) were purchased from Sino-British SIPPR/BK Laboratory Animals (Shanghai, China). Animals were housed at 22°C under a 12-h light/dark cycle, with free access to water and standard rodent chow (Peng et al., 2007). The use and care of animals were in compliance with institutional guidelines for health and care of experimental animals. Ani hydrochloride (purity 95%) was purchased from Fu-Ma Chemical & Engineering Company (Hangzhou, China). Methyllycaconitine (MLA) citrate was purchased from Sigma-Aldrich (St. Louis, MO, USA). 17β-E_2_ was purchased from Mei-Lun Biology Company (Dalian, China).

### Ovariectomy Surgery

Female mice were bilaterally ovariectomized as previously described (Sun et al., 2018). Briefly, mice were anesthetized with 10% chloral hydrate (0.03 mL/kg, i.p.). The dorsal skin was shaved and sterilized. The ovaries were exteriorized with the associated fat pad and fallopian tubes *via* a midline dorsal skin and muscle layer incision at the right or left side of the vertebral column, and then the wounds were closed. During sham operation for ovariectomy (OVX) surgery, mice received the same operation except for isolation and removal of the ovaries.

### Preparation of CS Models

CS Models were prepared as previously described (Fan et al., 2016). In general, mice were anesthetized with a combination of ketamine (15 mg/kg, i.p.) and diazepam (0.15 mg/kg, i.p.) after 6-h fast, while rats were anesthetized with a combination of ketamine (10 mg/kg, i.p.) and diazepam (0.1 mg/kg, i.p.) after an overnight fast. The animals were fixed in prone position, with hind limbs (2 and 4.5 cm from the ankles up for mice and rats respectively) compressed by 20 kg weights for 5 h.

### Serum Biochemical Assays

Serum K^+^ and glucose levels in mice were measured with a Hitachi 7600-120 automated chemistry analyzer (Hitachi, Tokyo, Japan). Mice serum insulin and E_2_ levels were determined by ELISA according to the manufacturer’s instructions (Shanghai Enzyme-linked Biotechnology, Shanghai, China). Homeostasis model assessment of insulin resistance (HOMA-IR) index = fasting serum insulin (mIU/L) × fasting serum glucose (mmol/L)/22.5. Quantitative insulin sensitivity check index (QUICKI) = 1/[log(fasting serum glucose)+log(fasting serum insulin)].

### Blood Pressure Measurement

Systolic blood pressure (SBP), diastolic blood pressure (DBP), mean blood pressure (MBP), and heart rate (HR) were continuously recorded as previously described with minor modifications (Liu et al., 2012; Yu et al., 2013). Briefly, rats were anesthetized with a combination of ketamine (50 mg/kg, i.p.) and diazepam (5 mg/kg, i.p.). A polyethylene catheter was inserted into the ascending aorta through left common carotid artery for blood pressure measurement and another polyethylene catheter was inserted into left external jugular vein for drug injection. After a two-day recovery period, blood pressure and HR were determined under a conscious condition.

### Experimental Protocols

#### Experiment 1: Gender Difference of Serum K^+^, E_2_, Insulin Sensitivity, and Mortality in Mice With CS

Female mice were randomly divided into normal group (n = 6) and CS model group (n = 6, mice received compression). Twelve male mice were divided and treated similarly. Blood samples were collected from vena cava at 6 h after decompression, and serum K^+^, E_2_, insulin, and glucose levels were measured, as well as in “normal” groups. CS models were established in another group of mice (n = 20 per gender). Survival time was monitored for 24 h after decompression.

#### Experiment 2: Influences of Ani on Serum K^+^, E_2_, and Insulin Sensitivity in Mice With CS

Male mice were randomly divided into five groups (n = 6 per group): 1) normal: mice received normal saline (i.p.); 2) CS model: mice received normal saline (i.p.); 3) MLA (10 mg/kg); 4) Ani (28 mg/kg); 5) Ani (28 mg/kg) + MLA (10 mg/kg). CS models were established in groups 2 to 5. Ani was given i.p. at 30 min before decompression, and MLA was given i.p. 30 min earlier. Blood samples were collected from vena cava at 6 h after decompression, and serum K^+^, E_2_, insulin, and glucose levels were measured, as well as in “normal” group.

#### Experiment 3: Influences of OVX and Ani/E_2_ Treatment on Serum K^+^, E_2_, and Insulin Sensitivity in Mice With CS

Female mice were randomly divided into four groups (n = 6 per group) 2 weeks after OVX surgery: 1) normal: mice received normal saline (i.p.); 2) CS model: mice received normal saline (i.p.); 3) Ani (28 mg/kg); 4) E_2_ (100 mg/kg). CS models were established in the later three groups. Four groups (n = 6 per group) of female mice were treated similarly 2 weeks after sham operation for OVX surgery. Ani and E_2_ were given i.p. at 30 min before decompression. Blood samples were collected from vena cava at 6 h after decompression, and serum K^+^, E_2_, insulin, and glucose levels were measured, as well as in “normal” group.

#### Experiment 4: Effects of Ani and E_2_ on Mortality in Mice With CS

Female mice were randomly divided into three groups (n = 20 per group) 2 weeks after OVX surgery: 1) CS model: mice received normal saline (i.p.); 2) Ani (28 mg/kg); 3) E_2_ (100 mg/kg). CS models were established in all the groups. Three groups (n = 20 per group) of female mice were treated similarly 2 weeks after sham operation for OVX surgery. Ani and E_2_ were given i.p. at 30 min before decompression. Survival time was monitored for 24 h after decompression.

#### Experiment 5: Influences of Ani on Blood Pressure in Rats With CS

Male rats were randomly divided into five groups (n = 6 per group): 1) normal: rats received normal saline (i.p.); 2) CS model: rats received normal saline (i.p.); 3) MLA (7 mg/kg); 4) Ani (20 mg/kg); 5) Ani (20 mg/kg) + MLA (7 mg/kg). After catheterization and recovery for 2 d, CS models were established in groups 2 to 5. Ani was injected i.p. at 30 min before decompression, and MLA was injected i.p. 30 min earlier. Blood pressure was monitored for 3.5 h since 30 min before decompression, as well as in “normal” group.

### Statistical Analysis

Data are shown as mean ± SD. T test or t’ test was used for data of experiments involving only two groups. ANOVA was used for data of experiments involving more than two groups, followed by Bonferroni test or Games-Howell test. Kaplan-Meier analysis was used for survival time analysis, followed by a log-rank test. Survival rate between two groups were analyzed with Fisher’s exact test. *P* < 0.05 was considered statistically significant. Analyses were performed using SPSS 21.0 (SPSS, Inc., Chicago, IL, USA).

## Results

### Gender Difference of Serum K^+^, E_2_, and Mortality in Mice With CS

**Figure 1:**
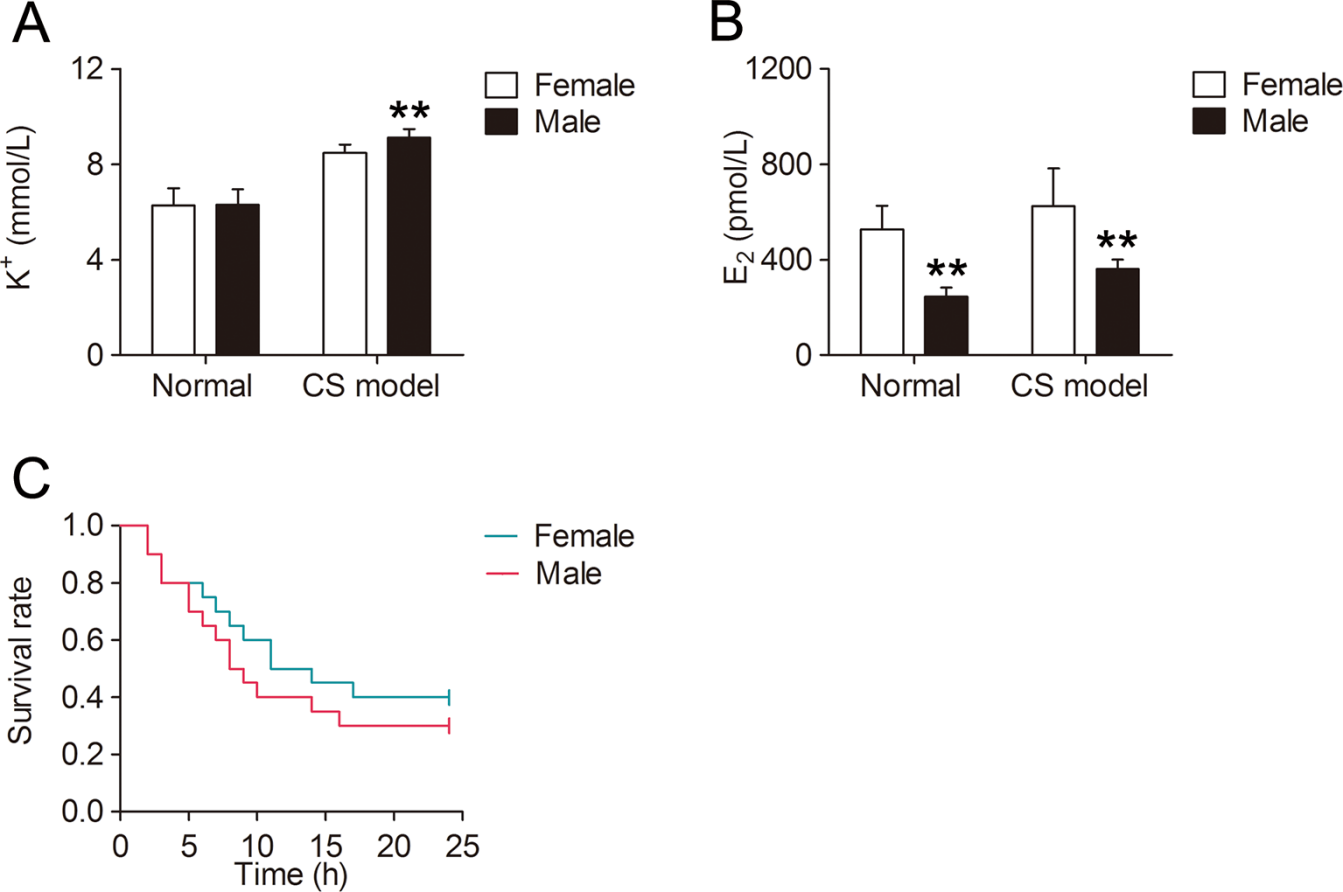
Gender difference of serum K^+^, E_2_, and mortality in mice with crush syndrome (CS). Blood samples of mice with CS were collected at 6 h after decompression (n = 6 per group). Serum K^+^ level was significantly lower in female CS models than that in male **(A)**. Serum E_2_ level was much higher in both female and male CS models than that in normal mice **(B)**. There was no significant difference in survival time between female and male CS models (C, n = 20 per group). ^**^ *P* < 0.01 vs. female.

Serum K^+^ level at 6 h after decompression was significantly higher in male mice (+7.6%, *P* < 0.01, **Figure 1A**) compared with that in female mice, and there was no difference in serum K^+^ between normal female and male mice. Male mice had significantly lower serum E_2_ level than female mice whether compressed or not. Furthermore, serum E_2_ was much higher in both female and male CS models (+18.7%, +47.1% respectively, **Figure 1B**) than that in normal mice. The mortality within 24 h was 60% and 70% for female and male CS models respectively (**Figure 1C**).

### Ani Decreases Serum K^+^ and Increases Serum E_2_ in Mice With CS

**Figure 2:**
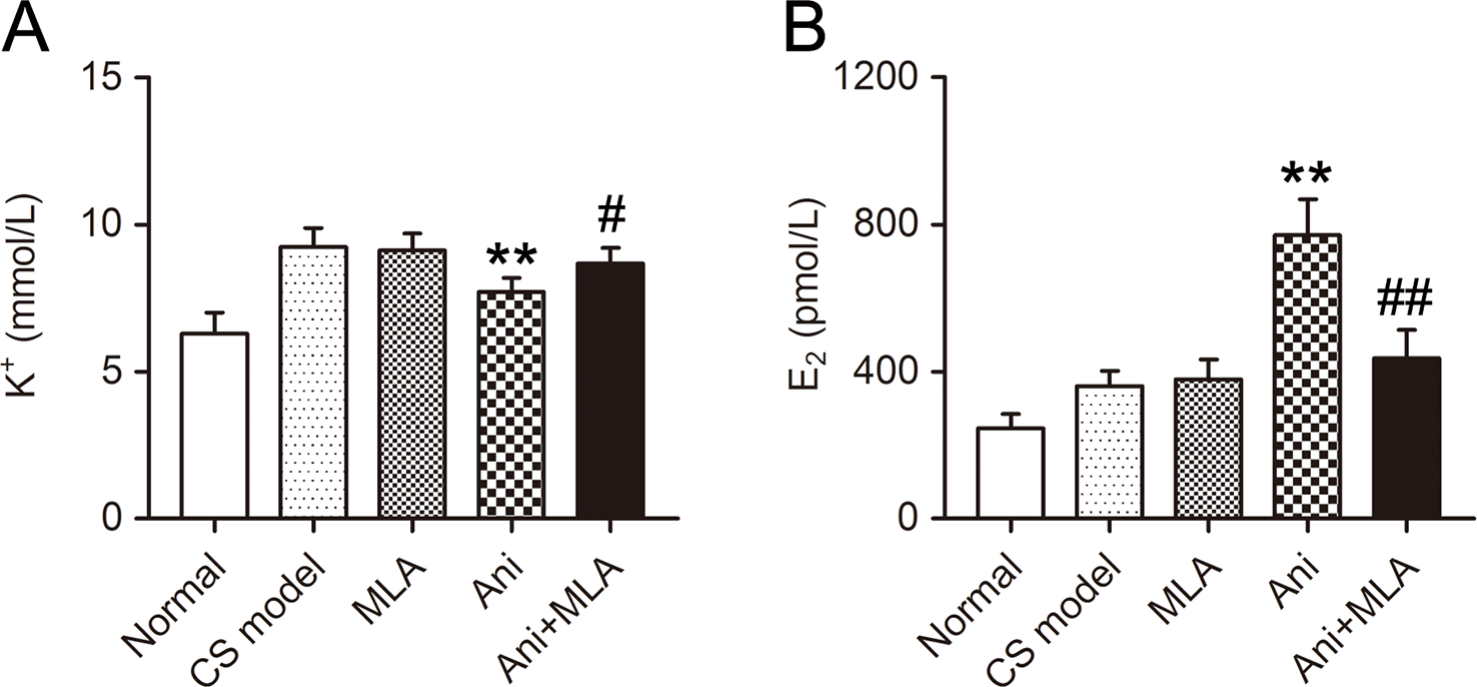
Anisodamine (Ani) decreases serum K^+^ and increases serum E_2_ in male mice with crush syndrome (CS). Ani (28 mg/kg, i.p.) was administrated at 30 min before decompression in mice with CS, and methyllycaconitine (MLA) (10 mg/kg, i.p.) was given 30 min earlier. Blood samples were collected at 6 h after decompression. Ani decreased serum K^+^ level after decompression, and MLA significantly counteracted such effect of Ani **(A)**. Ani increased serum E_2_ level after decompression, and MLA significantly counteracted such effect of Ani **(B)**. N = 6 per group. ^**^ *P* < 0.01 vs. CS model. ^#^ *P* < 0.05, ^##^ *P* < 0.01 vs. Ani.

Serum K^+^ was significantly lower in Ani (−16.5%, *P* < 0.01, **Figure 2A**) treated mice compared with that in model controls, and significantly higher in Ani + MLA group compared with that in Ani group (+12.4%, *P* < 0.05). Serum E_2_ was significantly higher in Ani (+113.7%, *P* < 0.01, **Figure 2B**) treated mice compared with that in model controls, and significantly lower in Ani + MLA group compared with that in Ani group (−43.4%, *P* < 0.05).

### Influences of OVX and Ani/E_2_ Treatment on Serum K^+^ and E_2_ in Mice With CS

**Figure 3:**
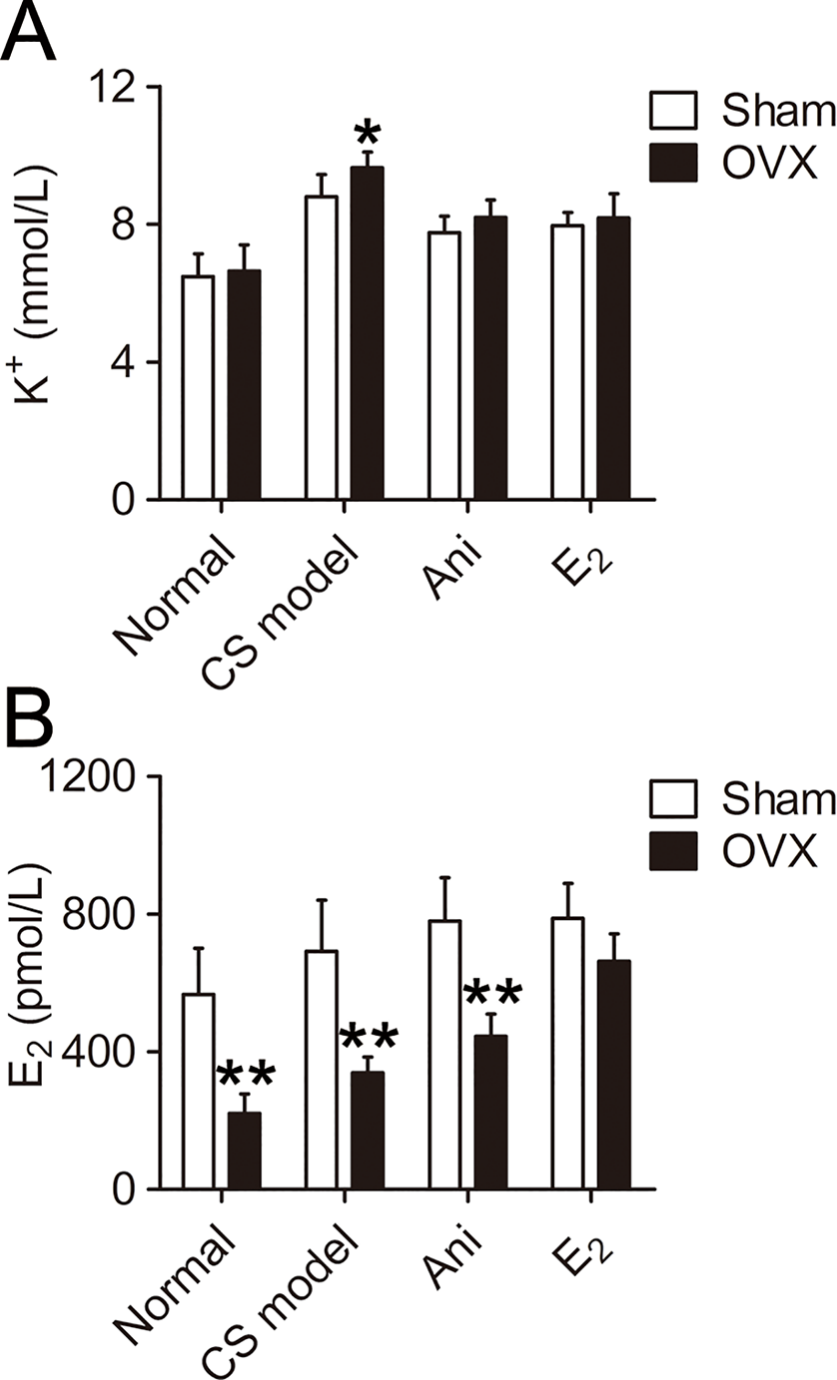
Influences of ovariectomy (OVX) and anisodamine (Ani)/E_2_ treatment on serum K^+^ and E_2_ in mice with crush syndrome (CS). Ani (28 mg/kg, i.p.) or E_2_ (100 mg/kg, i.p.) was administrated at 30 min before decompression in mice with CS 2 weeks after OVX surgery or its sham operation. Blood samples were collected at 6 h after decompression. OVX increases serum K^+^ level after decompression. Both Ani and E_2_ eliminated OVX-induced difference in serum K^+^ and decreased serum K^+^ in CS models **(A)**. OVX decreased serum E_2_ level, and E_2_ treatment narrowed such difference. Both Ani and E_2_ increased serum E_2_ in CS models received OVX surgery **(B)**. N = 6 per group. *P < 0.05, **P < 0.01 vs. sham. Sham, sham operation for OVX surgery.

Serum K^+^ was significantly higher in CS models received OVX surgery (+9.5%, *P* < 0.05, **Figure 3A**) compared with that in those received sham operation for OVX surgery. Serum K^+^ decreased in CS models received OVX surgery or its sham operation through Ani (−14.9%, −11.8% respectively) or E_2_ (−15.1%, −9.5% respectively) treatment. OVX surgery did not affect serum K^+^ in normal mice. OVX surgery-induced difference in serum K^+^ was eliminated by both Ani and E_2_ treatment. Serum E_2_ was significantly lower in mice received OVX surgery compared with that in those received sham operation for OVX surgery, except for those received E_2_ treatment (**Figure 3B**). Serum E_2_ increased in CS models received OVX surgery or its sham operation through Ani (+31.5%, +12.6% respectively) or E_2_ (+95.4%, +13.8% respectively) treatment.

### Ani and E_2_ Decreases Mortality in Mice With CS

**Figure 4:**
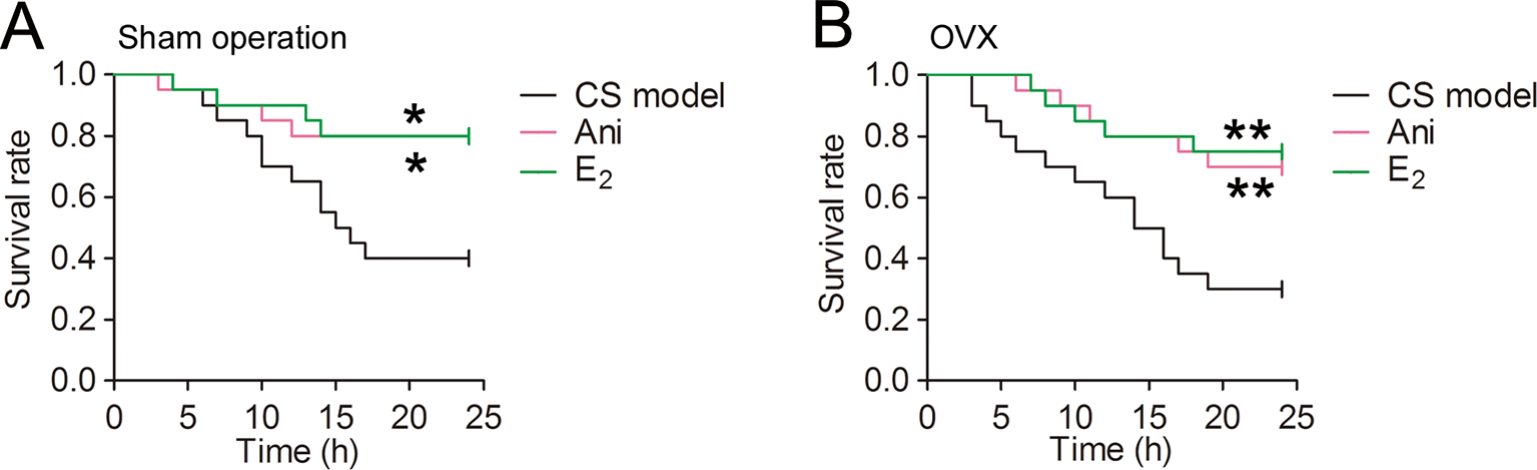
Anisodamine (Ani) and E_2_ decreases mortality in mice with crush syndrome (CS). Ani (28 mg/kg, i.p.) or E_2_ (100 mg/kg, i.p.) was administrated at 30 min before decompression in mice with CS 2 weeks after ovariectomy (OVX) surgery or its sham operation. Both Ani and E_2_ prolonged survival time in mice with CS received OVX surgery **(B)** or its sham operation **(A)**. N = 20 per group. ^*^ *P* < 0.05, ^**^ *P* < 0.01 vs. CS model.

The mortality within 24 h was much lower in Ani and E_2_ groups compared with that in CS model controls received OVX surgery (30% and 25% vs. 70%, **Figure 4B**) or its sham operation (20% and 20% vs. 60%, **Figure 4A**). Survival time was significantly longer in Ani and E_2_ groups compared with that in CS model controls received OVX surgery (log-rank testing χ^2^ = 6.84, *P* = 0.009, χ^2^ = 8.00, *P* = 0.005 respectively) or its sham operation (log-rank testing χ^2^ = 5.65, *P* = 0.017, χ^2^ = 5.98, *P* = 0.014 respectively).

### Ani Increases Insulin Sensitivity in Mice With CS

**Figure 5:**
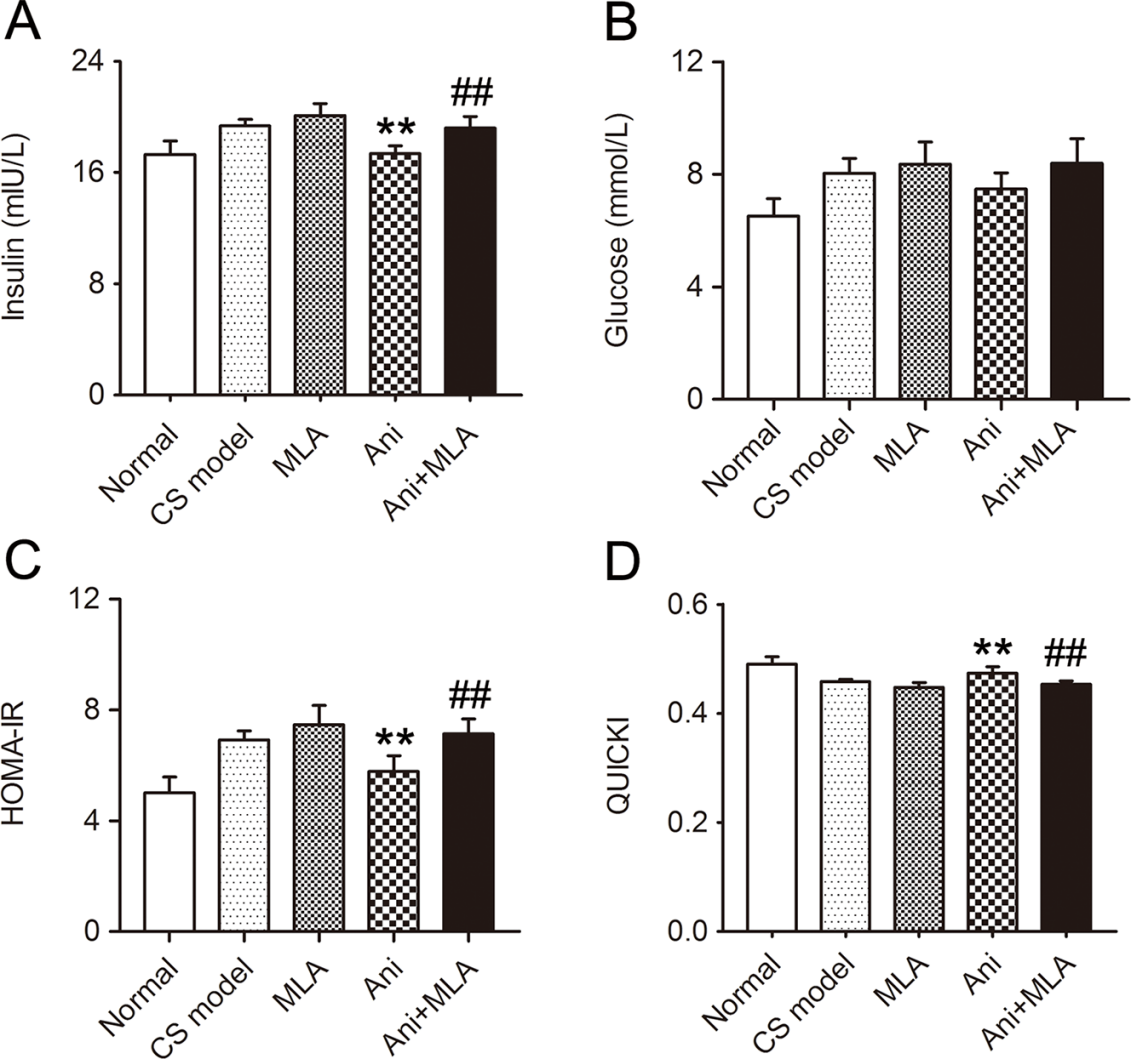
Anisodamine (Ani) increases insulin sensitivity in male mice with crush syndrome (CS). Ani (28 mg/kg, i.p.) was administrated at 30 min before decompression in mice with CS, and methyllycaconitine (MLA) (10 mg/kg, i.p.) was given 30 min earlier. Blood samples were collected at 6 h after decompression. Ani reduced serum insulin level **(A)** and homeostasis model assessment of insulin resistance index **(C)**, elevated quantitative insulin sensitivity check index **(D)** after decompression, but had no significant effect on serum glucose level **(B)**. MLA significantly counteracted such effects of Ani. N = 6 per group. **P < 0.01 vs. CS model. ^##^ *P* < 0.01 vs. Ani.

Treatment with Ani significantly reduced serum insulin (*P* < 0.01, **Figure 5A**) and HOMA-IR (*P* < 0.01, **Figure 5C**), elevated QUICKI index (*P* < 0.01, **Figure 5D**), and slightly reduced serum glucose level (**Figure 5B**) in mice with CS, indicating a higher insulin sensitivity in Ani treated mice. However, MLA attenuated the effect of Ani on insulin sensitivity, reflected by elevated serum insulin (*P* < 0.01), glucose, and HOMA-IR (*P* < 0.01), and reduced QUICKI index (*P* < 0.01).

### Gender Difference of Insulin Sensitivity in Mice With CS

**Figure 6:**
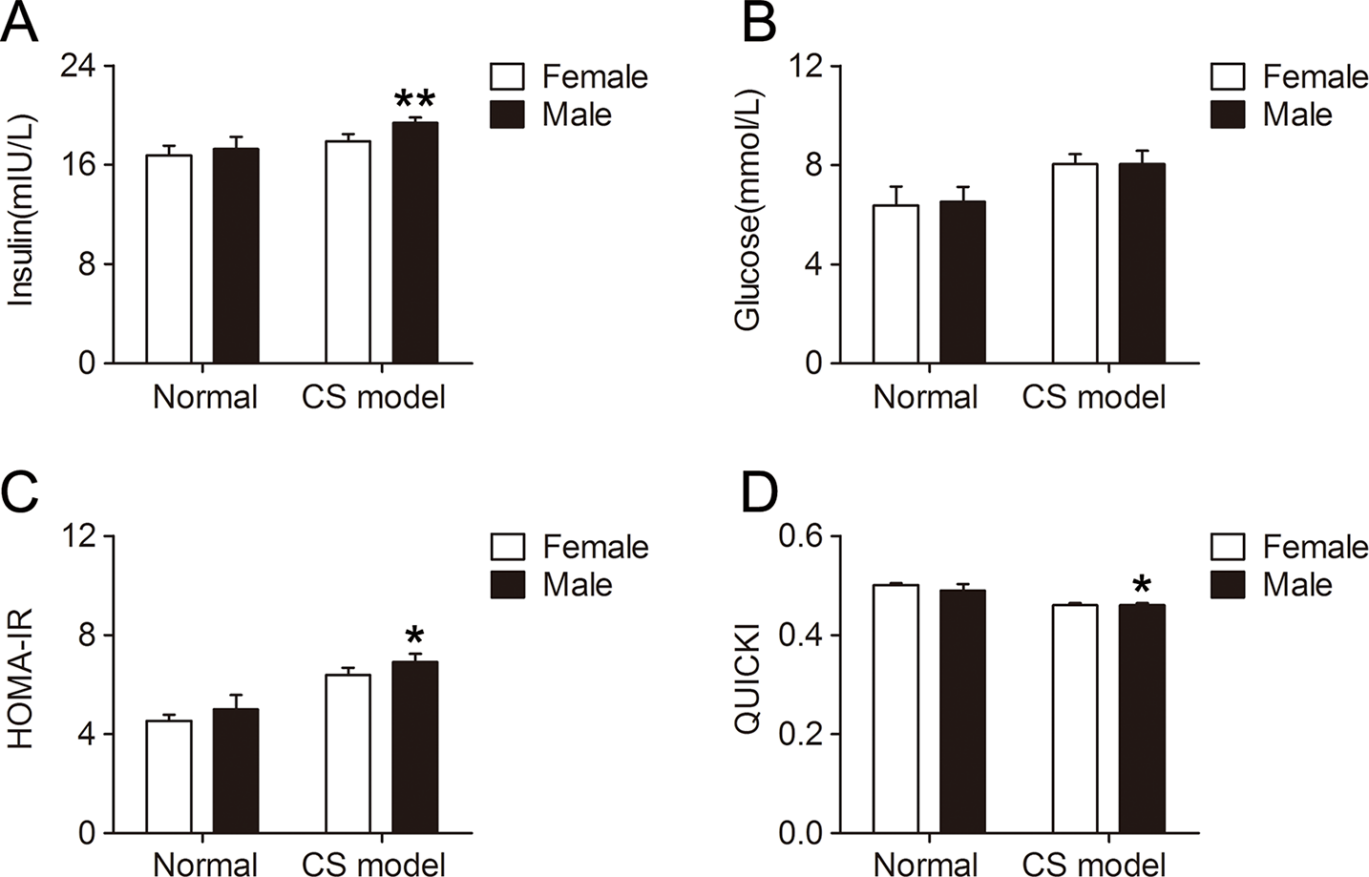
Gender difference of insulin sensitivity in mice with crush syndrome (CS). Blood samples of mice with CS were collected at 6 h after decompression. Female CS models exhibited lower serum insulin level **(A)** and homeostasis model assessment of insulin resistance index **(C)**, and higher quantitative insulin sensitivity check index **(D)** than that in male. Serum glucose level was not affected by gender in CS models **(B)**. N = 6 per group. *P < 0.05, **P < 0.01 vs. female.

Insulin sensitivity was significantly lower in male CS models compared with that in female CS models, reflected by higher serum insulin level (*P* < 0.01, **Figure 6A**) and HOMA-IR (*P* < 0.05, **Figure 6C**), and lower QUICKI index (*P* < 0.05, **Figure 6D**). Serum glucose level was not significantly affected by gender in CS models (**Figure 6B**). There was no difference in insulin sensitivity between normal female and male mice.

### Influences of OVX and Ani/E_2_ Treatment on Insulin Sensitivity in Mice With CS

**Figure 7:**
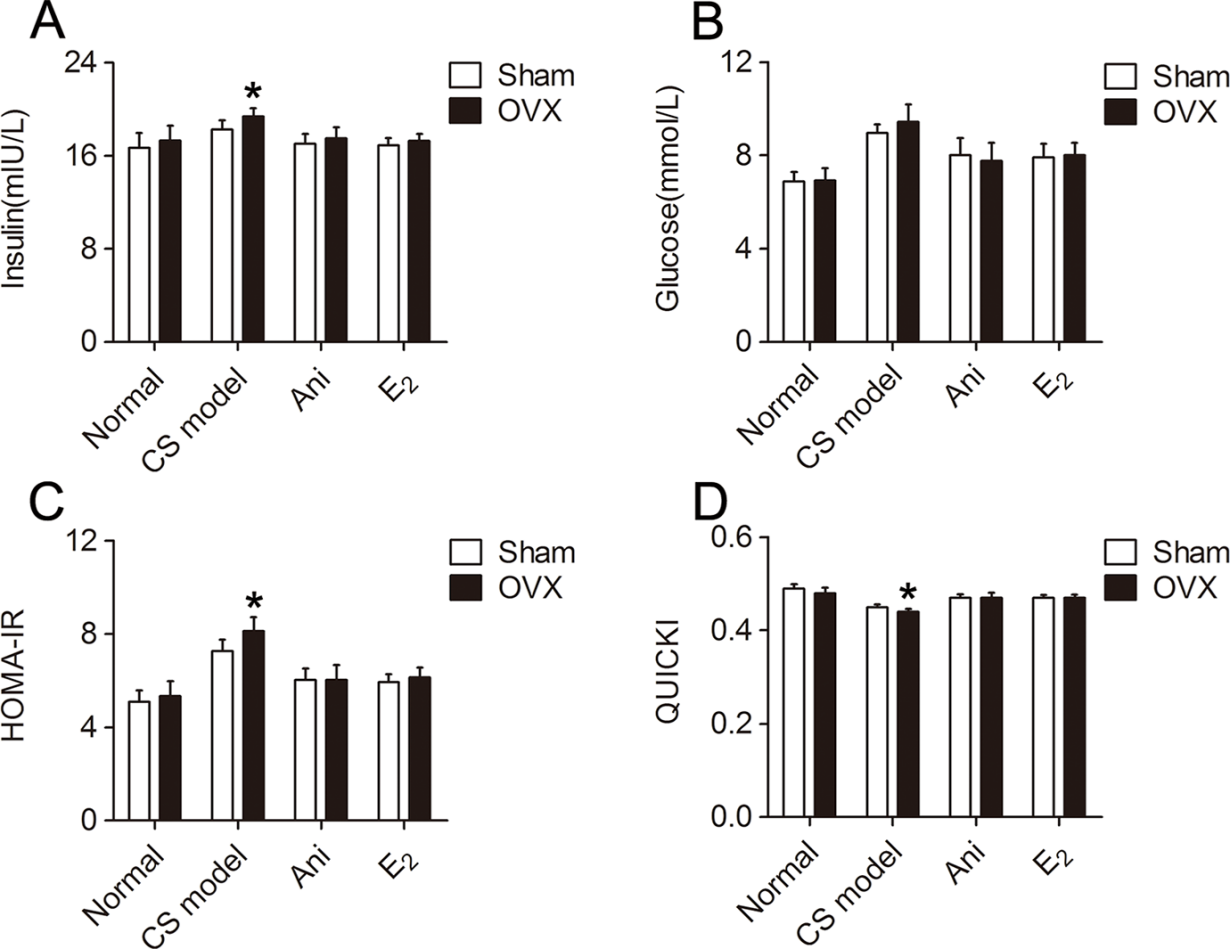
Influences of ovariectomy (OVX) and Anisodamine (Ani)/E_2_ treatment on insulin sensitivity in mice with crush syndrome (CS). Ani (28 mg/kg, i.p.) or E_2_ (100 mg/kg, i.p.) was administrated at 30 min before decompression in mice with CS 2 weeks after OVX surgery or its sham operation. Blood samples were collected at 6 h after decompression. OVX elevated serum insulin level **(A)** and homeostasis model assessment of insulin resistance index **(C)**, reduced quantitative insulin sensitivity check index **(D)** after decompression, but had no significant effect on serum glucose level **(B)**. Both Ani and E_2_ eliminated OVX-induced difference in insulin sensitivity and increased insulin sensitivity in CS models. N = 6 per group. *P < 0.05 vs. Sham, sham operation for OVX surgery.

Insulin sensitivity was significantly lower in CS models received OVX surgery compared with that in those received sham operation for OVX surgery, reflected by elevated serum insulin (*P* < 0.05, **Figure 7A**) and HOMA-IR (*P* < 0.05, **Figure 7C**), and reduced QUICKI index (*P* < 0.05, **Figure 7D**). Serum glucose level was not significantly affected by OVX surgery (**Figure 7B**). Insulin sensitivity increased in CS models received OVX surgery or its sham operation through Ani or E_2_ treatment, reflected by reduced serum insulin, glucose and HOMA-IR, and elevated QUICKI index. OVX surgery did not affect insulin sensitivity in normal mice. OVX surgery-induced difference in insulin sensitivity was eliminated by both Ani and E_2_ treatment.

### Ani Increases Blood Pressure in Rats With CS

**Figure 8:**
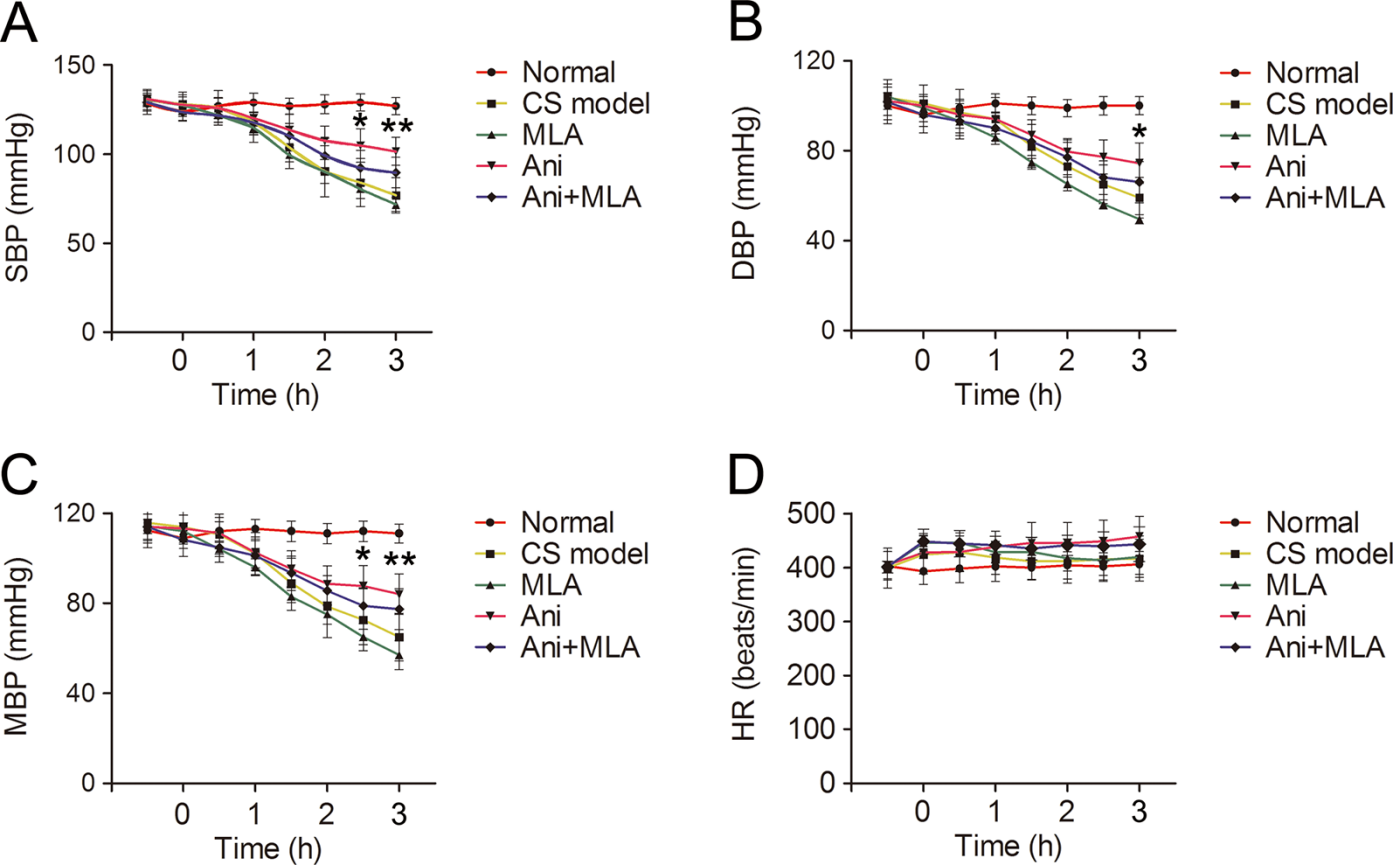
Anisodamine (Ani) increases blood pressure in rats with crush syndrome (CS). Ani (20 mg/kg, i.p.) was administrated at 30 min before decompression in rats with CS, and methyllycaconitine (MLA) (7 mg/kg, i.p.) was given 30 min earlier. Blood pressure was monitored for 3.5 h since 30 min before decompression. Systolic blood pressure (SBP), diastolic blood pressure (DBP), mean blood pressure (MBP) decreased gradually after decompression in rats with CS. Ani significantly increased SBP and MBP at 2.5 h after decompression and increased SBP **(A)**, DBP **(B)**, and MBP **(C)** at 3 h after decompression. MLA attenuated the effect of Ani on blood pressure. Heart rate (HR) was not affected by compression, decompression, or drug administration **(D)**. N = 6 per group. *P < 0.05, **P < 0.01 vs. CS model.

SBP, DBP, and MBP decreased gradually after decompression in rats with CS. Treatment with Ani significantly increased SBP (105 ± 9.4 vs. 84 ± 13 mmHg, *P* < 0.05, **Figure 8A**) and MBP (88 ± 9.3 vs. 73 ± 14 mmHg, *P* < 0.05, **Figure 8C**) at 2.5 h after decompression and increased SBP (102 ± 7.9 vs. 77 ± 9.9 mmHg, *P* < 0.01), DBP (74 ± 9.0 vs. 60 ± 9.0 mmHg, *P* < 0.05, **Figure 8B**) and MBP (84 ± 9.6 vs. 65 ± 11 mmHg, *P* < 0.01) at 3 h after decompression compared with that in model controls. MLA attenuated the effect of Ani on blood pressure. HR was not affected by compression, decompression, or drug administration (**Figure 8D**).

## Discussion

Hyperkalemia, occurring shortly after decompression, is a major cause of on-site death in CS (Ashkenazi et al., 2005). Sever and his research team reported that serum potassium is the most significant predictor of dialysis needs in the CS victims recued during the first 3 days (Sever et al., 2003). Though there is no gender difference in serum potassium in the healthy, it’s puzzling that both severity and frequency of hyperkalemia is higher in male CS victims, represented as higher serum potassium at admission (5.0% higher than female) and more dialysis needs; however, there is no difference in mortality between female and male (16.1% vs. 14.3%) victims (Sever et al., 2002; Sever et al., 2003; Sever et al., 2004). Consistent with these studies, we observed no gender differences in serum potassium in normal mice, and slightly higher serum potassium in male CS mice (7.6% higher than female), which was not high enough to cause a gender difference in on-site mortality. Furthermore, OVX was performed to remove the main source of endogenous E_2_, the major female sex hormone, in female mice. As expected, serum E_2_ was dramatically decreased after OVX, and serum potassium was higher in OVX mice after decompression compared to that in sham-operated mice. All the above-findings indicated the important role of E_2_ in gender difference of serum potassium during CS.

As known, stress can down-regulate the hypothalamic-pituitary-gonadal axis and subsequent production of endogenous sex hormones (Toufexis et al., 2014; Rasmusson et al., 2017). Interestingly, our results demonstrated that serum E_2_ was increased in both female and male CS mice with similar values, 98.42 pmol/L for female and 115.48 pmol/L for male. Coincidentally, serum E_2_ was increased in all CS mice received OVX or its sham operation with similar values, 125.35 pmol/L for sham operation and 117.72 pmol/L for OVX. Treatment with E_2_ was reported to decrease plasma potassium in OVX rats (Zheng et al., 2006). Normally, quantities of serum E_2_, estrogen receptor (ERα and ERβ), and G protein-coupled ER (GPER) in tissue are in a homeostasis, therefore, gonads-sourced E_2_ could only alleviate the increase of serum potassium within limits. Although serum potassium was lower in CS female mice, with far higher serum E_2_ than that in male or OVX mice, it was still dramatically higher than normal level. From the above, the increased part of serum E_2_ might originate from sources outside of gonads, such as adipose tissue, and act as a defense against sudden increase of serum potassium in CS.

Ani is widely used clinically for varieties of shock treatment in China, especially septic shock with fewer and less severe adverse effects compared with atropine (Li et al., 1999). Our previous studies showed that Ani could decrease on-site mortality in CS through modest reduction (−10%∼-20%) of serum potassium, and such effect was mediated by indirect activation of α7nAChR (Fan et al., 2016), which was verified with MLA, selective antagonist of α7nAChR, in the present study. PNU-282987, selective a7nAChR agonist, has been reported to increase serum E_2_ level and ERα and ERβ expression in OVX rats (Ma et al., 2015). We found that Ani might increase serum E_2_ in CS mice and MLA might counteract such effect of Ani. Treatment with Ani increased serum E_2_, and decreased serum potassium and on-site mortality in all CS mice received OVX or its sham operation. Serum E_2_ was reported to have a negative correlation with major arrhythmic cardiovascular events in patients with arrhythmogenic right ventricular cardiomyopathy/dysplasia that may lead to sudden cardiac death (Akdis et al., 2017). E_2_ could also prevent hyperkalemia-induced Ca^2+^ loading and hypercontracture in cardiomyocytes, which might exert cardioprotective effects during hyperkalemic cardioplegia (Jovanovic et al., 1998). We found that exogenous supplement of E_2_ exerted similar influence to Ani on serum potassium and on-site mortality. Disagreed with the result of Ma et al. that serum E_2_ was even higher in PNU-282987-treated OVX rats than that in sham-operated rats (Ma et al., 2015), a great gap still existed in serum E_2_ between sham-operated and OVX mice after Ani treatment; however, difference in serum potassium had been eliminated by Ani. Herein, decline of serum potassium in CS mainly depends on E_2_ originating from sources outside of gonads. E_2_ was reported to increase expression of α7nAChR in the brain and kidney of animals (Miller et al., 1982; Miller et al., 1984; Centeno et al., 2006; el-Mas et al., 2011). In addition, E_2_ and other ER agonists could increase ERα, ERβ, and GPER expression respectively (Pisolato et al., 2016; Wang et al., 2019). Taken together, there might be a crosstalk between α7nAChR and E_2_, as well as ERs, to regulate the expression of each other, which is the basis for Ani to ameliorate hyperkalemia and decrease on-site mortality in CS.

Enhancement of insulin sensitivity may decrease serum potassium, accompanying reduction of serum glucose. We previously proved that activation of α7nAChR decreases serum potassium in CS through elevation of insulin sensitivity, during which insulin downstream signaling molecules, such as phosphoinositide 3-kinase, mammalian target of rapamycin, and signal transducer and activator of transcription 3, were involved. Inhibition of the above-mentioned signaling molecules could decrease extracellular potassium (Fan et al., 2016). E_2_ has been reported to enhance insulin sensitivity and ameliorate insulin resistance in lots of experiments (Park et al., 2017; Qiu et al., 2018; Torres et al., 2018; Yan et al., 2019). In the present study, insulin sensitivity after decompression was lower in male and OVX female mice compared to the relative controls. Both Ani and exogenous supplement of E_2_ could increase insulin sensitivity after decompression and eliminate the difference between sham-operated and OVX mice. In addition, E_2_ could also stimulate activity and expression of Na/K-ATPase (Obradovic et al., 2014; Obradovic et al., 2015), which pumps sodium out of cells while pumping potassium into cells and plays an important role in insulin-induced decline of serum potassium (Chibalin et al., 2001; Al-Khalili et al., 2004). Therefore, Ani could decrease serum potassium during CS through E_2_-based activation of insulin signaling pathway.

We also examined the influence of Ani on hypovolemic shock in CS rats shortly after decompression in this study. Treatment with Ani could alleviate hypotension in CS rats induced by long-sustained compression, which could be attenuated by MLA, without influences on HR. Our previous study showed that Ani could increase blood pressure and decrease mortality in rats with hemorrhagic shock, which indirectly proved the causality between elevation of blood pressure and decline of mortality associated with hypovolemic shock during CS. Besides hyperkalemia-induced cardiac arrest, E_2_ could also exert neuroprotective effects after hypovolemic cardiac arrest (Semenas et al., 2011). Therefore, activation of α7nAChR with Ani could benefit both hyperkalemia and hypovolemic shock in CS through participation of E_2_.

## Conclusion

**Figure 9:**
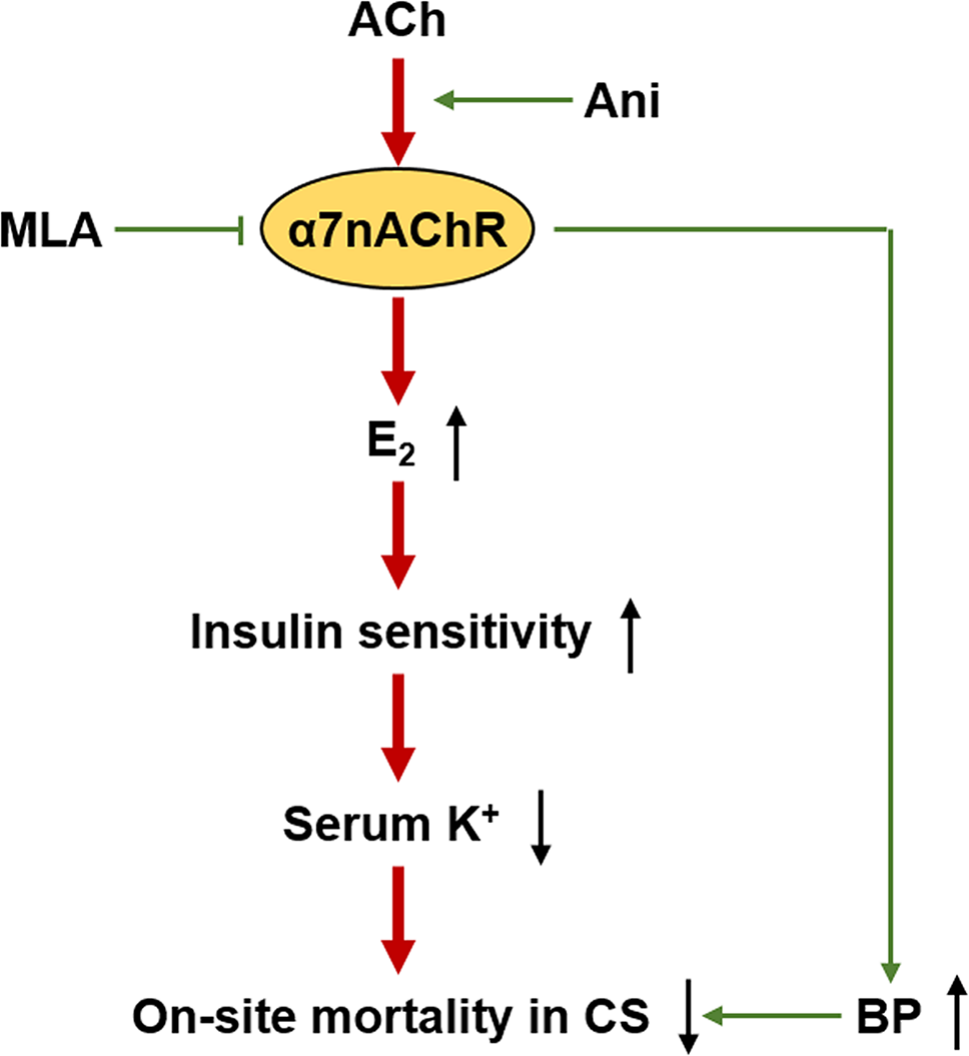
Proposed mechanism for anisodamine to decrease on-site mortality in crush syndrome. ACh, acetylcholine; BP, blood pressure.

This study demonstrates that activation of α7nAChR with Ani could ameliorate hyperkalemia during CS through E_2_-induced enhancement of insulin sensitivity, and thus to decrease on-site mortality (**Figure 9**). Ani could also alleviate hypovolemic shock in CS, during which the study about effect of E_2_ is still required. Moreover, Ani and E_2_ are encouraging drugs for on-site remedy of CS, which needs further investigations.

## Limitations

In the present study, effect of Ani on serum E_2_ in CS was well proved, as well as the effect of E_2_ on serum potassium and even on-site mortality. However, participation of ERs (ERα, ERβ, and GPER) in this process was not verified. Therefore, examination of ER expression and application of ER knockout mice is still required in further investigations.

## Data Availability Statement

The raw data supporting the conclusions of this manuscript will be made available by the authors, without undue reservation, to any qualified researcher.

## Ethics Statement

The animal study was reviewed and approved by Ethics Committee of Second Military Medical University.

## Author Contributions

J-GY and XL designed the study and experiments. J-GY, B-SF, and J-MG performed the experiments. Y-JS and Y-YH analyzed the data. J-GY and B-SF wrote the paper.

## Funding

This work was supported by Grants from the National Natural Science Foundation of China (81773726), National Science and Technology Major Project (2018ZX09J18110-003-001), and Innovation Cultivating Foundation of 6th Medical Center, PLA General Hospital (CXPY201829).

## Conflict of Interest

The authors declare that the research was conducted in the absence of any commercial or financial relationships that could be construed as a potential conflict of interest.
